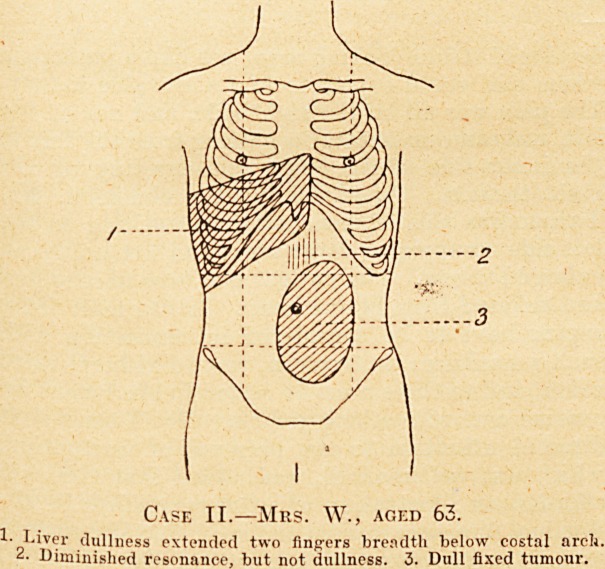# Two Cases of Hydatid of the Liver

**Published:** 1907-01-12

**Authors:** Leonard A. Bidwell

**Affiliations:** Surgeon West London Hospital, Dean of the Post-Graduate College


### Hospital Clinics.

### TWO CASES OF HYDATID OF THE LIVER.

By LEONARD A. BIDWELL, F.R.C.S.Eng., Surgeon West London Hospital, Dean of the Post-Graduate College.

In this country cases of hydatid of the liver are very uncommon, and it is curious that two such cases should have been under my care at the hospital within a couple of months. Both of them are unusual cases, and a description will be interesting.

Case I. was a young lady, aged 34, whom I saw with Dr. MacBurney, on May 2, on account of a large swelling in the upper part of the abdomen accompanied by general wasting. The following history was given. The patient had suffered from right pleurisy with effusion four years previously, and the chest had been aspirated; with this exception she had good health and remained well till November 1905, when she had an attack of jaundice, accompanied by abdominal pain and vomiting. After 14 days in bed the jaundice and pain disappeared, and she became convalescent. She did not, however, regain her strength, but got about till six weeks before I saw her, when she had another attack of jaundice, which was accompanied by vomiting and pain like indigestion. The pain and sickness went away after two or three days, but she began to grow weaker, so after three weeks

Case I.—Miss E. P., aged 34.

1. Apex beat. 2. Dull area. 3. Special prominent swelling, not absolutely dull.

she consulted Dr. MacBurney. He found that the abdomen was considerably enlarged, the breathing very embarrassed and the temperature about 100°. She was then kept in bed and on light diet, but the abdominal pains returned, were most marked on the right side, and gradually got worse. There was no further jaundice nor vomiting, but nausea was always present. There had been no rigor. The temperature was slightly elevated, the bowels were constipated. When I saw her she was considerably emaciated; there was a slight icteric tinge of the conjunctiva, but no jaundice. The temperature was 100°, the pulse 112, and the respiration 28. She suffered from dyspnœa on movement.

On examination of the abdomen there was a prominent swelling in the upper part occupying the epigastric and right hypochondriac regions, extending about four inches beyond the costal arch, and measuring about five inches across. It was dull on percussion, and there was a slight thrill in it. Below the swelling, the lower edge of the liver could be felt about one inch above the umbilicus. The swelling was only slightly tender and there was no redness of the skin. The rest of the abdomen moved fairly well, and was resonant. On examination of the chest the heart was found to be displaced outwards, the apex beat being one and a half inch outside the left nipple and in the sixth space; the sounds were normal. There was impaired resonance over the whole of the front of the right chest, and absolute dullness with loss of breath sound below the fourth cartilage. Posteriorly there was dullness and loss of breath sound from the angle of the scapula downwards. On the left side there was impaired resonance over the base behind.

Now here we had a case in which there was some swelling pushing down the liver and pushing up the lung; it was, therefore, situated in the subphrenic space, and we had to consider what its nature could be. The commonest form of swelling in the subphrenic space is an abscess, and the elevation of temperature in this case was in favour of this view; we then had to consider what are the commonest causes of abscess in the subphrenic space; of course an abscess in this place may arise in connection with disease of any viscus which is situated in the upper part of the abdomen, or even may track up from the lower part of the abdomen. The most common causes of subphrenic abscess are, first, rupture of a gastric or duodenal ulcer, where the neighbourhood of the ulcer has been shut off by adhesions prior to the rupture; secondly, it may follow suppuration in the gall-bladder or ducts, and is then secondary to acute cholecystitis; thirdly, it may be secondary to suppuration in the kidney; fourthly, to an abscess arising in connection with an ulcer of the colon; fifthly, in consequence of acute suppurative pancreatitis; and finally in connection with appendix suppuration. Now in the case before us the history was unlike that of a rupture of an ulcer of the stomach or duodenum. Thus there was no sudden onset, and the symptoms were completely relieved after the first attack; moreover the duration of the case for six weeks was rather too long for so localised a collection of fluid. With regard to the second cause, namely suppuration in the gall-bladder or ducts, everything seemed to point to this as the cause. We had a history of the onset of the first attack being abdominal pain, jaundice and vomiting—a pretty good history of gall-stones; the second phase of the illness commenced in a similar way, and the swelling coming on after a rise of temperature was suggestive of suppuration. The course of events seemed to have been—the engagement of a gall-stone in a duct causing inflammation of the duct, and consequently jaundice, followed after the second engagement by suppuration. The third cause of abscess, namely suppuration in the kidney, was negatived by the absence of any urinary symptoms, and of any previous attack of renal pain, since most of the cases of subphrenic abscess due to kidney suppuration are due to calculus, and symptoms of that condition have usually existed beforehand. The abscess in connection with ulcer of the colon is a rare condition and almost impossible to diagnose. Usually there are symptoms of ulcerative colitis, but these may be absent and symptoms of peritonitis may be the only sign; at least this was so in the one case which I have had. The fifth cause, namely acute suppurative pancreatitis, is also rather an obscure condition to diagnose, but this was very much in my mind when considering this case; the acuteness of the pain, the vomiting, which was profuse, and the emaciation, were greatly in favour of this obscure disease; there were, however, no other signs, such as fatty stools. Finally, abscess in connection with appendicitis was considered, as I am familiar with it. I have described to you a case which I had seen during an acute attack of appendicitis, and in which I found a subphrenic abscess some weeks afterwards without any abscess having formed in the region of the cæcum. Since then I have seen another case in which a subphrenic abscess formed secondarily to an abscess in the region of the cæcum. In the case which we are considering, the onset of the case was unlike that of acute appendicitis, and there never had been any signs of any inflammation of the appendix.

Finally, then, my diagnosis was a subphrenic abscess, due to acute cholecystitis, to acute pancreatitis, or to appendicitis; my final opinion was in favour of gall-stones as the original cause, but, of course, I am too old a hand to give a definite diagnosis before opening the abdomen. I was right about the subphrenic abscess, but I was wrong as to the cause of it.

I opened the abdomen over the most prominent part of the swelling by an incision through the outer fibres of the right rectus muscle. On opening the peritoneum a bluish-looking mass presented, which at first rather looked like a cyst-wall, and I thought that I had to deal with a pancreatic cyst. On enlarging my incision I found that the bluish mass was in reality the liver, which was tightly stretched over a resistant mass. On examining further, the peritoneum was quite healthy below the liver, and the stomach and other organs normal; above the liver, between it and the dome of the diaphragm, were dense adhesions, which, of course, were what I expected to find in a subphrenic abscess, the liver being pushed downwards three or four inches. I then put a sponge over the peritoneal incision and decided to explore the subphrenic region through an intercostal space. A large-sized aspirating needle was inserted through the seventh intercostal space in the anterior axillary line, and some pus was drawn off. An incision was then made over the site of the puncture, and $1\frac{1}{2}$ inch of the seventh rib was excised and the pleura opened. The dome of the diaphragm then bulged into the wound, and this was sutured all round to the parietal pleura for the space of about an inch. An incision was then made into the dome of the diaphragm, and a quantity of pus evacuated, followed by an immense number of hydatid cysts. A very large drainage tube was then inserted and stitched in position. The thoracic wound was closed, and the abdominal incision was then united by three layers of sutures in the ordinary way and sealed up with collodion.

There is little to mention in the further progress of the case; the abdominal wound healed perfectly, but cysts continued to be discharged in large quantities through the drainage-tube. Three days after the operation the right lung began to expand, the area of resonance advanced, and the dyspnœa improved. The drainage-tube was removed after three weeks, although the cysts were still being discharged. She left the hospital convalescent in another fortnight, but the wound was not healed, and cysts were still discharged at intervals. Her general condition, however, was excellent. I saw her again a few weeks later; she had gained flesh, but cysts still continued to escape, so I started injection of thymol. After another month the wound had healed, and the patient is now well.

Now with regard to the line of treatment adopted. First it is essential to make a preliminary abdominal section to ascertain what is the precise cause of the condition, and to make certain of the position of the abscess or cyst. After having done this it is best to drain the abscess through the chest wall so that there should be no fouling of the peritoneal cavity. The abdominal incision, however, should not be closed, as in cases of difficulty in finding an abscess in the subphrenic space it is useful to have a finger in the abdomen when passing an aspirator very deeply towards the liver.

After resecting the rib and opening the pleural cavity, stitching the parietal pleura to the dome of the pleura effectually shuts off the pleural cavity and prevents any pleurisy. Lastly, no attempt should be made actively to scrape these cysts, as the walls may be very thin in parts, and any violent scraping might cause perforation into the abdominal cavity. The after progress of these cases is always very tedious, as the discharge will continue until all the daughter cysts are evacuated. The destruction of the cysts may be hastened by injection of thymol, as was done in this case.

Now, ought the case to have been diagnosed? It is difficult to judge oneself, and so I am inclined to think that it could not be. The usual symptoms of hydatid cyst are a painless swelling of long duration, absence of febrile temperature, or of any effect on the general health, and of jaundice or vomiting. The only symptom of a hydatid cyst in this case was the thrill which was felt.

One word against a preliminary aspiration for diagnostic purposes. This should never be done, as it is impossible to say what organ may be damaged by accident, and had it been done in this case the peritoneum and liver would have been perforated. Moreover, the diagnostic result would have been *nil*, for after my aspiration only pus escaped, and this on being examined microscopically was found to contain no hydatid element, but gave a pure culture of streptococci.

The second case was a woman aged 63, whom I saw in July last. She had enjoyed good health and had worked hard as a charwoman. For twenty years she had noticed in the abdomen a lump which was situated below the umbilicus on the left side. In April she had a severe attack of jaundice, which followed colicky pain in the abdomen, and then passed off in a few weeks; after this she became emaciated and complained of severe pains in the back. When I saw her the abdomen was consider-

Case II.—Mrs. W., aged 63.

1. Liver dullness extended two fingers breadth below costal arch. 2. Diminished resonance, but not dullness. 3. Dull fixed tumour.

ably distended, and there was a large tumour, the size of a football, occupying the umbilical, left lumbar, and inguinal regions, its upper border being situated one inch above the umbilicus. The tumour appeared to be fixed, was dull on percussion, and there was a well-marked thrill. Its position rather suggested an ovarian cyst, so a vaginal examination was made, but the tumour appeared to have no connection with the uterus or appendages. The liver was enlarged and extended about two finger breadths below the ribs; there was an interval of two or three inches between the liver dullness and that of the tumour; this area was resonant, but on deep pressure considerable resistance was felt. The abdomen was not tender on palpation. The area of liver dullness extended upwards to the fourth rib in front.

There was no sign of ascites; both flanks were resonant. The other organs were healthy. The temperature was normal, and the pulse was 70.

Here was a case presenting several difficulties in diagnosis. In the first place we had a cystic tumour which was situated principally on the left side and below the umbillicus, and which had existed for twenty years; it had grown slowly, but more rapidly lately. The first diagnosis was an ovarian cyst, but against this was the freedom of the tumour from the uterus and appendages; this freedom is sometimes found in old-standing ovarian cysts with long pedicles. The second point against an ovarian cyst was the fixed condition, and the third the apparent continuation of the cyst behind the resonant area above the umbilicus. This latter sign seemed to point to some cyst arising in the lesser cavity of the peritoneum and extending downwards. Now the most common cyst in this position is one in connection with the pancreas, and this was carefully considered. Another cystic swelling in this region is a hydronephrosis, but in such a case the swelling nearly always extends into the loin, which did not occur in this case. I therefore excluded kidney, and was rather in favour of a pancreatic cyst. Now pancreatic cysts do not give rise to the same symptoms of pain and emaciation that occur in chronic pancreatitis, and they are often practically painless swellings; they do not, however, last so long as twenty years, and this was the main point against my diagnosis. Then, again, we had the history of the colicky pain, followed by intense jaundice. Was this connected with the tumour in the lower half of the abdomen, or was it quite independent of it? If dependent on the tumour, it could practically only be caused by pressure, and this seemed hardly likely when the greater part of the tumour was in the lower half of the abdomen. I therefore rather inclined to the view that the pain and jaundice were independent symptoms and were most probably due to gall-stones. Against this was the absence of any enlargement of the gall-bladder or of any tenderness on pressure just below the tip of the eighth rib. The enlargement of the area of liver dullness, however, was quite consistent with the gall-stone trouble. The absence of any temperature, and the comparative freedom from pain, put aside the question of cholecystitis and of abscess formation, but even if these had been present it would not have accounted for the tumour below. I therefore thought that there were two distinct conditions present: first an old ovarian cyst, and secondly gall-stones.

As the biliary symptoms were the most important, I made an exploratory incision through the fibres of the right rectus about one inch outside of the middle line at the level of the umbilicus, intending to extend the incision upwards if gall-stones were present, but wishing to explore the lower swelling thoroughly to see if it had any connection with the liver. On opening the abdomen the right edge of the lower swelling was exposed and found to be cystic; this was palpated upwards, and was traced up to the under surface of the liver. My incision was then prolonged upwards, and the under surface of the liver examined. The gall-bladder and ducts were found to be healthy, but the common duct was adherent to the swelling. The condition was then diagnosed as hydatid cyst, and a hole was torn in the gastro-colic omentum opening up the lesser peritoneal sac. The cyst-wall was then exposed and the parietal peritoneum was then stitched to the cyst-wall round an area of about two square inches. A free incision in the area was then made into the cyst, which was found to contain closely packed hydatid cysts, the size of small peas, with practically no fluid between them. They would not flow out, so had to be evacuated with a blunt spoon. About two quarts of these cysts were evacuated and a large drainage-tube introduced. The rest of the abdomen now was closed in the ordinary way. The patient did well for the first few days but developed some lung trouble. She had dyspnœa dullness and absence of breath-sounds over the right base and fever. At first I suspected a subphrenic abscess, and put an exploring syringe into the chest without finding pus. The chest trouble, however, cleared up without any abscess formation, and the patient made a good recovery. The discharge of cysts, however, has continued up till now, although the patient has gained flesh and the abdominal tumour has entirely disappeared. I have recently commenced treatment with thymol, and this, I hope, will be effectual.

These two cases show how difficult the diagnosis of abdominal tumour often is, and they also show that hydatid cysts may produce much more severe symptoms than is usually expected of them. With cases of such rarity it is often long before we are able to put into practice the experience gained by mistakes in diagnosis.

## Figures and Tables

**Case I. f1:**
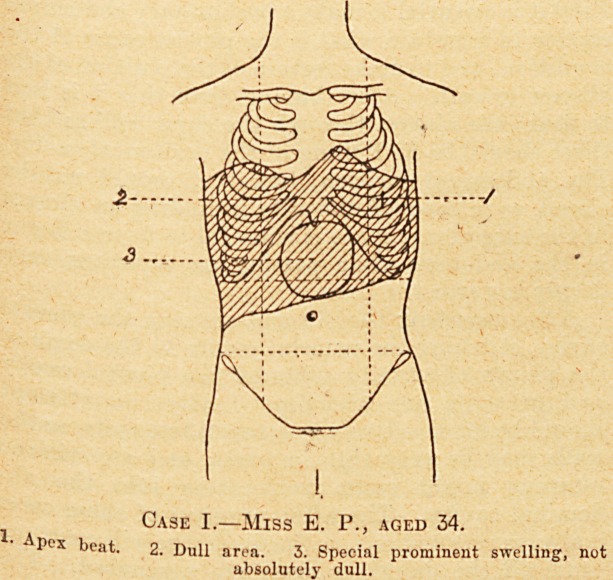


**Case II. f2:**